# Adjunctive brexpiprazole in patients with major depressive disorder who show minimal or partial response to antidepressant treatment: *post hoc* analysis of randomized controlled trials

**DOI:** 10.1093/ijnp/pyaf074

**Published:** 2025-10-07

**Authors:** Shivani Kapadia, Zhen Zhang, Ferhat Ardic, Mehul Patel, Michael E Thase, George I Papakostas

**Affiliations:** Medical Affairs, Otsuka Pharmaceutical Development & Commercialization Inc., Princeton, NJ, United States; Medical and Real-World Data Analytics, Otsuka Pharmaceutical Development & Commercialization Inc., Princeton, NJ, United States; Medical Affairs, H. Lundbeck A/S, Valby, Denmark; Medical Affairs, Otsuka Pharmaceutical Development & Commercialization Inc., Princeton, NJ, United States; Perelman School of Medicine, University of Pennsylvania and the Philadelphia Veterans Affairs Medical Center, Philadelphia, PA, United States; Department of Psychiatry, Clinical Trials Network and Institute, Massachusetts General Hospital, Harvard Medical School, Boston, MA, United States

**Keywords:** clinical trials, depression, subgroup analysis, treatment augmentation, treatment optimization

## Abstract

**Introduction:**

Many patients with major depressive disorder (MDD) have <50% symptom reduction on antidepressant treatment, and may benefit from an adjunctive atypical antipsychotic. This *post hoc* analysis aimed to investigate the efficacy and safety of adjunctive brexpiprazole in patients with minimal (>0% to <25%) and partial (≥25% to <50%) response to antidepressant treatment.

**Methods:**

Data were pooled from three international, randomized, double-blind, placebo-controlled, Phase 3 trials. Adult outpatients with MDD and inadequate response to antidepressant treatments were enrolled. Patients were stratified *post hoc* into minimal and partial response subgroups based on their response over an 8-week prospective antidepressant treatment period. Adjunctive brexpiprazole 2–3 mg/day (versus adjunctive placebo) was investigated in a 6-week randomized treatment period. Efficacy was assessed using the Montgomery–Åsberg Depression Rating Scale (MADRS) and the Clinical Global Impressions – Severity of illness (CGI-S). Safety was assessed by treatment-emergent adverse events (TEAEs).

**Results:**

In patients with minimal response to antidepressant treatment (*n* = 663), least squares (LS) mean (SE) MADRS total score change over the randomized treatment period was −8.8 (0.3) points for antidepressant + brexpiprazole and −6.3 (0.3) points for antidepressant + placebo; the LS mean difference at Week 6 was −2.47 (95% CI, −3.38 to −1.55); *P*<.001; Cohen’s *d*, 0.41. In patients with partial response to antidepressant treatment (*n* = 235), corresponding changes were −6.4 (0.5) and −4.9 (0.5) points; LS mean difference, −1.53 (−2.94 to −0.11); *P* = .035; Cohen’s *d*, 0.28. CGI-S results aligned with MADRS results. In patients with minimal response to antidepressant treatment, the incidence of TEAEs was 196/328 (59.8%) for antidepressant + brexpiprazole and 160/335 (47.8%) for antidepressant + placebo. In patients with partial response to antidepressant treatment, corresponding values were 63/115 (54.8%) and 49/120 (40.8%).

**Conclusions:**

Adjunctive brexpiprazole may be efficacious in MDD regardless of whether patients show minimal or partial response to antidepressant treatment.

**Trial registration:**

*Post hoc* analysis of NCT01360645, NCT01360632, NCT02196506 (ClinicalTrials.gov).

**Significance Statement**

Depression is a common mental disorder associated with a constant feeling of sadness or lack of interest in daily life. Treatment with antidepressant medication can help to improve symptoms of depression. However, the amount of improvement varies from person to person. For people who still have symptoms on antidepressants, one option is to add brexpiprazole. Brexpiprazole is a medication that can help to improve symptoms of depression when taken with an antidepressant. Our study used data from clinical trials to look at the effects of brexpiprazole in people with either “minimal” or “partial” improvement on antidepressant. We found that, in both groups, people who added brexpiprazole had a greater improvement in symptoms than people who added a placebo (dummy drug). This means that adding brexpiprazole may help people with depression regardless of the amount of improvement they had on antidepressant alone.

## INTRODUCTION

Depressive disorders such as major depressive disorder (MDD) are prevalent and burdensome conditions, ranking second of all diseases (after lower back pain) in terms of years of healthy life lost due to disability.^1^^,^^2^ Antidepressant treatment is a first-line treatment recommendation for MDD,^3–5^ supported by a large evidence base.^6^ However, an analysis of 182 randomized clinical trials found that approximately half of patients with MDD have <50% symptom reduction on antidepressant treatment.^7^ Unresolved symptoms of depression can have a detrimental impact on functioning, quality of life, well-being, and productivity, and should not be left untreated.^8^^,^^9^

For patients with unresolved symptoms of depression on optimized antidepressant treatment, next treatment steps include switching to another antidepressant or augmenting the antidepressant, among other options.^3–5^ Traditionally, switching antidepressant is preferred for patients with minimal or non-response, often defined as <25% symptom reduction from baseline,^10–14^ since it is assumed that these patients may show a larger benefit on a different therapy. Augmentation is traditionally preferred for patients with partial response to antidepressant treatment, often defined as 25%–50% symptom reduction from baseline.^10-14^ The logic behind this approach is that the initial antidepressant response will be maintained by continuing the same antidepressant, and enhanced by augmentation.^11^^,^^12^^,^^15^ However, while randomized clinical trials support the efficacy of certain adjunctive therapies for unresolved symptoms of depression,^16^^,^^17^ there is little-to-no clinical evidence exploring treatment efficacy in patients with minimal response versus partial response to antidepressant treatment.^12^^,^^13^ Contrary to treatment traditions, it could be hypothesized that augmentation may have a larger effect in patients with minimal response to antidepressant treatment, due to the greater scope for improvement.^12^

Atypical antipsychotics are perhaps the most extensively studied adjunctive treatment options in MDD.^4^^,^^16^^,^^17^ Across a large Phase 3 clinical program in MDD, adjunctive brexpiprazole was efficacious and well tolerated in patients with inadequate response to antidepressant treatments, which was broadly defined as <50% symptom reduction.^18–21^ The aim of this *post hoc* analysis, using pooled data from three randomized clinical trials in MDD, was to investigate the efficacy and safety of adjunctive brexpiprazole in patients with minimal response to antidepressant treatment and with partial response to antidepressant treatment.

## METHODS

The three trials described in this *post hoc* analysis were registered at ClinicalTrials.gov (identifiers: NCT01360645, NCT01360632, NCT02196506), approved by appropriate institutional review boards or independent ethics committees (previously listed^22^), and conducted in accordance with the Declaration of Helsinki, the International Conference on Harmonization Good Clinical Practice guideline, and local regulatory requirements. Written informed consent was provided by all participants prior to enrolment.

### Trial Design and Patients

The three trials were similarly designed, short-term, randomized, double-blind, placebo-controlled, fixed-dose, Phase 3 trials of adjunctive brexpiprazole in patients with MDD and inadequate response to antidepressant treatments.^18–20^ The trials were conducted in the period 2011–2016 in Europe, Russia, and North America. Detailed trial designs have been previously published.^18–20^

In brief, the trials enrolled male and female outpatients aged 18–65 years with a diagnosis of MDD (single episode or recurrent; Diagnostic and Statistical Manual of Mental Disorders, Fourth Edition, Text Revision [DSM-IV-TR] criteria^23^) and a current depressive episode that had lasted for ≥8 weeks. Additionally, patients must have trialed at least one but no more than three antidepressants during the current episode, at adequate dose and duration, and have had an inadequate response to these antidepressants defined as <50% improvement according to the Massachusetts General Hospital Antidepressant Treatment Response Questionnaire (self-reported).^24^ Patients with any psychotic symptomatology or any other protocol-specified DSM-IV-TR Axis I or II mental disorder were excluded.

Following screening, patients entered an 8-week prospective treatment period in which they received a new antidepressant (selective serotonin reuptake inhibitor or serotonin–norepinephrine reuptake inhibitor) selected by the investigator from the following list: duloxetine, escitalopram, fluoxetine, paroxetine controlled release, sertraline, or venlafaxine extended release. This new antidepressant was administered in an open-label manner, together with adjunctive placebo, which was blinded to patients but not to investigators. The intention of the prospective treatment period was to identify patients with inadequate response to this additional antidepressant, who would then be eligible to continue into the randomized treatment period. In all three trials (following protocol amendments in two trials), inadequate response was defined as <50% reduction in Hamilton Depression Rating Scale^25^ total score from the start to the end of prospective treatment and score ≥ 14 at the end of prospective treatment; Clinical Global Impressions – Improvement^26^ score ≥ 3 (“minimally improved”) at Weeks 2, 4, 6, and 8 of prospective treatment; and < 50% reduction in Montgomery–Åsberg Depression Rating Scale (MADRS)^27^ total score from the start to Weeks 2, 4, 6, and 8 of prospective treatment. Thus, following the prospective treatment period, patients had shown inadequate response to 2–4 antidepressants in total during the current episode.

In the randomized treatment period, eligible patients received double-blind adjunctive brexpiprazole or adjunctive placebo for 6 weeks, while continuing on the same antidepressant. In two trials, patients were randomized 1:1 to brexpiprazole 2 mg/day or placebo; in the other trial, patients were randomized 1:1:1 to brexpiprazole 1 mg/day, brexpiprazole 3 mg/day, or placebo. Brexpiprazole was titrated, starting at 0.5 mg/day, up to 1 mg/day after 1 week, and to the allocated fixed-dose after 2 weeks.

### Outcome Measures

The clinician-rated MADRS was the predefined primary efficacy measure in each trial.^27^ The MADRS is a validated and reliable measure of the severity of ten depressive symptoms that are sensitive to treatment changes. MADRS total score can range from 0 (least severe depression) to 60 (most severe depression). During the randomized treatment period, the MADRS was completed at baseline and at weekly intervals by qualified and certified raters.

The clinician-rated Clinical Global Impressions – Severity of illness (CGI-S)^26^ scale was a secondary efficacy measure, also completed at baseline and at weekly intervals in the randomized treatment period. The CGI-S comprises a single item used to assess the overall severity of mental illness (in this case, depression) from 1 (normal, not at all depressed) to 7 (among the most extremely depressed patients).

Among several standard safety measures, the incidence of patient-reported treatment-emergent adverse events (TEAEs) was determined, including individual TEAEs and TEAEs leading to discontinuation. Investigators enquired about TEAEs on a weekly basis during the randomized treatment period.

### Definition of Minimal and Partial Response to Antidepressant Treatment

For the present analysis, patients were stratified according to their degree of response to antidepressant treatment over the 8-week prospective treatment period, using similar definitions to those in previous analyses:^12^^,^^13^

- Minimal response to antidepressant treatment was defined as >0% to <25% improvement in MADRS total score from the start to the end of the prospective treatment period, hereafter abbreviated to “0%–25%” response.
- Partial response to antidepressant treatment was defined as ≥25% to <50% improvement in MADRS total score from the start to the end of the prospective treatment period, hereafter abbreviated to “25%–50%” response.

Patients whose MADRS total score did not improve (ie, ≤0% improvement) on antidepressant treatment over the 8-week prospective treatment period were not considered in this analysis, as in previous analyses.^12^^,^^13^ Similarly, patients with “full” response to antidepressant treatment (≥50% improvement in MADRS total score over the prospective treatment period) were excluded; these patients were not randomized (see “Trial design and patients”) and would not typically require adjunctive treatment in clinical practice.

### Data Analysis

Analyses were performed in the efficacy sample, composed of patients randomized per final protocols who received at least one dose of double-blind medication and who had a baseline and at least one post-baseline MADRS total evaluation in the randomized treatment period. Patient data from the three trials were pooled for brexpiprazole 2 or 3 mg/day, representing the approved target (2 mg) and maximum (3 mg) doses for the adjunctive treatment of MDD in the US.^28^ A corresponding pooled placebo group was also created. Given that the 3 mg dose is not approved for MDD in some countries,^29^ an alternate analysis was performed of brexpiprazole 2 mg versus placebo using pooled patient data from the two trials that investigated the 2 mg dose;^18^^,^^20^ this analysis is presented in the online supplement.

Baseline values were the last values obtained prior to randomization. Patient baseline demographic and clinical characteristics were summarized using descriptive statistics.

Efficacy was analyzed by least squares mean changes from baseline in MADRS total score and CGI-S score using observed data in a mixed model for repeated measures (MMRM) with factors of treatment, visit, site nested in trial, baseline, interaction of treatment with visit, and interaction of baseline with visit. An unstructured covariance was used by default. Between-group comparisons were tested at a nominal 0.05 level (two-sided), with no adjustment for multiplicity. Cohen’s *d* effect sizes were determined.^30^

Safety was analyzed using descriptive statistics to summarize the incidence of TEAEs, TEAEs leading to discontinuation, extrapyramidal symptom-related TEAEs, and TEAEs with incidence >5% for antidepressant + brexpiprazole and greater than antidepressant + placebo.

All analyses used SAS Enterprise Guide 8.2 (SAS Institute Inc, Cary, NC, USA).

## RESULTS

### Patients

Across the three trials, the efficacy sample comprised 1162 patients. The antidepressants assigned to these patients are listed in Table 1. Over 8 weeks in the prospective antidepressant treatment period, 264 (22.7%) patients did not improve (≤0%) on antidepressant, 663 (57.1%) patients had minimal (0%–25%) response to antidepressant treatment, and 235 (20.2%) patients had partial (25%–50%) response to antidepressant treatment.

**Table 1:** Baseline characteristics and assigned ADTs for ADT + brexpiprazole 2–3 mg and ADT + placebo, stratified by response to ADT.

	Minimal (0%–25%) response to ADT subgroup		Partial (25%–50%) response to ADT subgroup	
Characteristic, *n* (%) unless otherwise stated	ADT + placebo (*n* = 335)	ADT + brexpiprazole 2–3 mg (*n* = 328)	ADT + placebo (*n* = 120)	ADT + brexpiprazole 2–3 mg (*n* = 115)
**Age (years), mean (SD)**	44.5 (12.3)	44.1 (11.7)	44.3 (11.3)	44.3 (11.4)
**Sex**				
** Female**	238 (71.0)	228 (69.5)	78 (65.0)	88 (76.5)
** Male**	97 (29.0)	100 (30.5)	42 (35.0)	27 (23.5)
**BMI (kg/m^2^), mean (SD)**	29.2 (6.9)	29.6 (6.9)	29.1 (7.3)	29.6 (7.2)
**Race**				
** White**	285 (85.1)	291 (88.7)	104 (86.7)	101 (87.8)
** Other^a^**	50 (14.9)	37 (11.3)	16 (13.3)	14 (12.2)
**Duration of current episode (months), mean (SD)**	16.0 (32.8)	15.6 (26.6)	15.8 (27.9)	13.5 (16.2)
**Number of lifetime episodes, mean (SD)**	3.5 (2.7)	3.4 (2.7)	3.4 (2.2)	3.3 (2.0)
**Number of prior ADTs at screening^b^**				
** 1**	277 (82.7)	272 (84.0)	95 (79.2)	84 (74.3)
** 2**	54 (16.1)	44 (13.6)	20 (16.7)	26 (23.0)
** 3**	4 (1.2)	8 (2.5)	5 (4.2)	3 (2.7)
**MADRS total score, mean (SD)**	26.6 (4.4)	26.5 (4.3)	21.1 (3.3)	21.6 (3.6)
**CGI-S score, mean (SD)**	4.2 (0.6)	4.2 (0.6)	3.9 (0.6)	3.8 (0.5)
**Assigned ADT**				
** Escitalopram**	68 (20.3)	62 (18.9)	18 (15.0)	25 (21.7)
** Fluoxetine**	52 (15.5)	54 (16.5)	11 (9.2)	8 (7.0)
** Paroxetine CR**	32 (9.6)	33 (10.1)	14 (11.7)	16 (13.9)
** Sertraline**	48 (14.3)	44 (13.4)	26 (21.7)	20 (17.4)
** Duloxetine**	72 (21.5)	80 (24.4)	28 (23.3)	26 (22.6)
** Venlafaxine XR**	63 (18.8)	55 (16.8)	23 (19.2)	20 (17.4)

a Including American Indian or Alaska Native, Asian, Black or African American, Native Hawaiian or Other Pacific Islander, and other non-specified (US Census Bureau classifications).

b Number of prior ADTs at screening was missing for 6 patients.

Abbreviations: ADT, antidepressant treatment; BMI, body mass index; CGI-S, Clinical Global Impressions – Severity of illness; CR, controlled release; MADRS, Montgomery–Åsberg Depression Rating Scale; SD, standard deviation; XR, extended release.

### Minimal (0%–25%) Response to Antidepressant Subgroup

Following randomization, the minimal response to antidepressant treatment subgroup comprised 328 patients on antidepressant + brexpiprazole 2–3 mg/day and 335 patients on antidepressant + placebo. The 6-week randomized treatment period was completed by 310 (94.5%) patients on antidepressant + brexpiprazole 2–3 mg/day and 320 (95.5%) patients on antidepressant + placebo.

At baseline (ie, the start of the randomized treatment period), mean (standard deviation [SD]) MADRS total score was 26.5 (4.3) for antidepressant + brexpiprazole 2–3 mg/day and 26.6 (4.4) for antidepressant + placebo. These scores had improved from 30.4 (4.4) and 30.5 (4.7), respectively, at the start of the 8-week prospective antidepressant treatment period.

The minimal response to antidepressant treatment subgroup was 70.3% (466/663) female, 29.7% (197/663) male, 86.9% (576/663) White, and 13.1% (87/663) other races (including American Indian or Alaska Native, Asian, Black or African American, Native Hawaiian or Other Pacific Islander, and other non-specified). Further demographic and clinical characteristics at baseline of the randomized treatment period are presented in Table 1, and were similar between treatments.

### Partial (25%–50%) Response to Antidepressant Subgroup

Following randomization, the partial response to antidepressant treatment subgroup comprised 115 patients on antidepressant + brexpiprazole 2–3 mg/day and 120 patients on antidepressant + placebo. The 6-week randomized treatment period was completed by 105 (91.3%) patients on antidepressant + brexpiprazole 2–3 mg/day and 116 (96.7%) patients on antidepressant + placebo.

At baseline (ie, the start of the randomized treatment period), mean (SD) MADRS total score was 21.6 (3.6) for antidepressant + brexpiprazole 2–3 mg/day and 21.1 (3.3) for antidepressant + placebo. These scores had improved from 31.3 (4.6) and 31.0 (4.1), respectively, at the start of the 8-week prospective antidepressant treatment period.

The partial response to antidepressant treatment subgroup was 70.6% (166/235) female, 29.4% (69/235) male, 87.2% (205/235) White, and 12.8% (30/235) other races. Further demographic and clinical characteristics at baseline of the randomized treatment period are presented in Table 1, and were similar between treatments.

Corresponding baseline data for the antidepressant + brexpiprazole 2 mg/day analyses are presented in the online supplement.

### Efficacy

#### Minimal (0%–25%) Response to Antidepressant Subgroup

**Figure 1:**
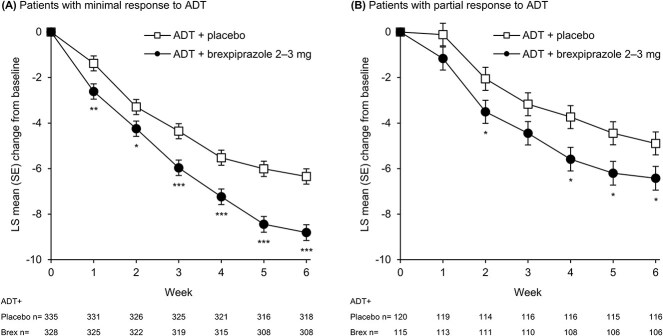
Mean MADRS total score change for ADT + brexpiprazole 2–3 mg versus ADT + placebo groups, stratified by response to ADT. ^*^P<.05, ^**^P<.01, ^***^P<.001 versus ADT + placebo; MMRM, observed cases. Mean baseline scores (ADT + placebo, ADT + brexpiprazole 2–3 mg): A) minimal response: 26.6, 26.5; B) partial response: 21.1, 21.6. Abbreviations: ADT, antidepressant treatment; Brex, brexpiprazole; LS, least squares; MADRS, Montgomery–Åsberg Depression Rating Scale; MMRM, mixed model for repeated measures; SE, standard error.

In patients with minimal response to antidepressant treatment, least squares (LS) mean (standard error [SE]) MADRS total score change from baseline to Week 6 was −8.8 (0.3) points for antidepressant + brexpiprazole 2–3 mg/day and −6.3 (0.3) points for antidepressant + placebo. The LS mean (95% confidence interval [CI]) treatment difference at Week 6 was −2.47 (−3.38 to −1.55), with *P*<.001 and effect size 0.41 in favor of antidepressant + brexpiprazole 2–3 mg/day. Considering other timepoints, antidepressant + brexpiprazole 2–3 mg/day was associated with greater improvement than antidepressant + placebo (*P*<.05) at all visits from Week 1, onwards (Figure 1A).

CGI-S results were supportive, with LS mean (SE) changes from baseline to Week 6 of −1.1 (0.0) for antidepressant + brexpiprazole 2–3 mg/day and −0.8 (0.0) for antidepressant + placebo, with an LS mean (95% CI) treatment difference of −0.25 (−0.36 to −0.13); *P*<.001 and effect size 0.33.

#### Partial (25%–50%) Response to Antidepressant Subgroup

In patients with partial response to antidepressant treatment, LS mean (SE) MADRS total score change from baseline to Week 6 was −6.4 (0.5) points for antidepressant + brexpiprazole 2–3 mg/day and −4.9 (0.5) points for antidepressant + placebo. The LS mean (95% CI) treatment difference at Week 6 was −1.53 (−2.94 to −0.11), with *P*=.035 and effect size 0.28 in favor of antidepressant + brexpiprazole 2–3 mg/day. Considering other timepoints, antidepressant + brexpiprazole 2–3 mg/day was associated with greater improvement than antidepressant + placebo (*P*<.05) at Week 2 and from Week 4, onwards (Figure 1B).

CGI-S results were supportive, with LS mean (SE) changes from baseline to Week 6 of −1.0 (0.1) for antidepressant + brexpiprazole 2–3 mg/day and −0.8 (0.1) for antidepressant + placebo, with an LS mean (95% CI) treatment difference of −0.21 (−0.40 to −0.01); *P* = .038 and effect size 0.27.

Corresponding efficacy data for the antidepressant + brexpiprazole 2 mg/day analyses also favored adjunctive brexpiprazole versus adjunctive placebo at Week 6 in patients with minimal and partial response, and are presented in the online supplement.

### Safety

#### Minimal (0%–25%) Response to Antidepressant Subgroup

In patients with minimal response to antidepressant treatment, the incidence of TEAEs was 196/328 (59.8%) for antidepressant + brexpiprazole 2–3 mg/day and 160/335 (47.8%) for antidepressant + placebo. TEAEs with incidence >5% for antidepressant + brexpiprazole 2–3 mg/day and greater than antidepressant + placebo were akathisia, increased weight, and restlessness; details are presented in Table 2.

**Table 2:** Summary of TEAEs for ADT + brexpiprazole 2–3 mg and ADT + placebo, stratified by response to ADT.

	Minimal (0%–25%) response to ADT subgroup		Partial (25%–50%) response to ADT subgroup	
Event, *n* (%)	ADT + placebo (*n* = 335)	ADT + brexpiprazole 2–3 mg (*n* = 328)	ADT + placebo (*n* = 120)	ADT + brexpiprazole 2–3 mg (*n* = 115)
**At least 1 TEAE**	160 (47.8)	196 (59.8)	49 (40.8)	63 (54.8)
**Discontinued due to adverse event**	3 (0.9)	9 (2.7)	0	2 (1.7)
**At least 1 EPS-related TEAE**	19 (5.7)	47 (14.3)	2 (1.7)	16 (13.9)
**TEAEs with incidence >5% for ADT + brexpiprazole and greater than ADT + placebo**				
** Akathisia**	13 (3.9)	33 (10.1)	0	10 (8.7)
** Headache**	22 (6.6)	14 (4.3)	3 (2.5)	7 (6.1)
** Increased weight**	7 (2.1)	18 (5.5)	0	7 (6.1)
** Nasopharyngitis**	9 (2.7)	7 (2.1)	3 (2.5)	6 (5.2)
** Restlessness**	2 (0.6)	18 (5.5)	0	5 (4.3)

Abbreviations: ADT, antidepressant treatment; EPS, extrapyramidal symptom; TEAE, treatment-emergent adverse event.

#### Partial (25%–50%) Response to Antidepressant Subgroup

In patients with partial response to antidepressant treatment, the incidence of TEAEs was 63/115 (54.8%) for antidepressant + brexpiprazole 2–3 mg/day and 49/120 (40.8%) for antidepressant + placebo. TEAEs with incidence >5% for antidepressant + brexpiprazole 2–3 mg/day and greater than antidepressant + placebo were akathisia, headache, increased weight, and nasopharyngitis; details are presented in Table 2.

Corresponding safety data for the antidepressant + brexpiprazole 2 mg/day analyses were similar to those of the antidepressant + brexpiprazole 2–3 mg/day analyses, and are presented in the online supplement.

## DISCUSSION

In this pooled analysis of clinical trial data, adjunctive brexpiprazole 2–3 mg was associated with greater improvement in depressive symptoms than adjunctive placebo in patients with MDD, regardless of whether they showed minimal (0%–25%) or partial (25%–50%) response to antidepressant treatment. This finding indicates that patients with minimal response to antidepressant treatment may benefit from augmenting, thereby providing an alternative to the current preference in clinical practice for switching antidepressant in these patients.^10-14^ This is particularly important given the diminishing effectiveness of monotherapy switches after two failed treatments, as seen in the landmark Sequenced Treatment Alternatives to Relieve Depression (STAR^*^D) study.^31^^,^^32^

Due to the potential for brexpiprazole to enhance the initial antidepressant treatment benefit, the present results suggest that augmentation with brexpiprazole could be considered as a next-step treatment option for all eligible patients with unresolved symptoms on antidepressant treatment, whether showing minimal or partial response to their antidepressant. Furthermore, in patients with minimal response to antidepressant treatment, adjunctive brexpiprazole showed greater improvement than adjunctive placebo at the first post-baseline visit (Week 1, and maintained at all weeks), suggesting that adjunctive brexpiprazole may provide rapid benefits in patients with a high level of unresolved symptoms. Given that early optimization of treatment is critical to help bring patients with MDD to full symptomatic and functional recovery,^33^ the decision to augment should be made as early as possible.

The mean change in MADRS total score from baseline to Week 6 with adjunctive brexpiprazole was numerically larger in patients with minimal response to antidepressant treatment (−8.8 points) than in patients with partial response to antidepressant treatment (−6.4 points), which is likely to reflect the higher baseline score in patients with minimal response (26.5–26.6 versus 21.1–21.6), thereby allowing greater scope for improvement. Similarly, a pooled analysis of adjunctive aripiprazole data showed a larger mean change at Week 6 in patients with minimal response versus partial response to antidepressant treatment.^12^ However, unlike adjunctive brexpiprazole, adjunctive aripiprazole was not different from adjunctive placebo with *P*<.05 at Week 6 in patients with partial response to antidepressant treatment.^12^ Thus, to the authors’ knowledge, adjunctive brexpiprazole is the only augmentation therapy that has been investigated, and shown efficacy, in patients with minimal response to antidepressant treatment and in patients with partial response to antidepressant treatment.

With regard to brexpiprazole dose, results were similar for the 2–3 mg and 2 mg analyses. Thus, the conclusion that adjunctive brexpiprazole is efficacious in patients with minimal or partial response to antidepressant treatment applies regardless of the different dose recommendations across countries.^28^^,^^29^

Although the present analysis did not consider specific depressive symptoms, it should be noted that even patients who do achieve response may experience unresolved symptoms of depression, such as anxiety and irritability.^34^ In prospective exploratory studies, patients with MDD and inadequate response to antidepressant treatment taking adjunctive brexpiprazole showed improvement in anxiety and irritability symptoms.^35^^,^^36^  *Post hoc* analyses support these observations, showing efficacy for antidepressant + brexpiprazole versus antidepressant + placebo on anxiety symptoms, as well as on depressive symptoms in patients with anxiety and irritability.^37-40^

In terms of safety, there were no notable differences in the incidence of TEAEs for adjunctive brexpiprazole when stratified by degree of response to antidepressant treatment. In patients with minimal and partial response to antidepressant treatment, adjunctive brexpiprazole was associated with a higher incidence of extrapyramidal symptoms (mostly akathisia) and weight gain than adjunctive placebo, as previously reported for the total pooled sample.^22^ Long-term safety analyses suggest that adjunctive brexpiprazole may be associated with moderate weight gain (approximately 4 kg on average over a year), and small changes in metabolic parameters and prolactin.^41-44^ Although no studies have compared the efficacy and safety of adding brexpiprazole versus switching antidepressant, a randomized trial showed a lower rate of treatment discontinuation for adjunctive aripiprazole versus switching to bupropion, despite a higher incidence of extrapyramidal symptoms and weight gain with aripiprazole.^45^

Strengths of this *post hoc* analysis are its exploration of a new hypothesis, and that similarly designed trials were pooled to obtain a large sample size that increased statistical power.^46^ Limitations include that the original trials were not designed or powered for minimal/partial response analyses, the *post hoc* analysis plan was not registered, and there were no adjustments for multiple testing. This increases the risk for Type I errors (false positives) and means that results should be confirmed in a prespecified analysis.^46^ The trials were only 6 weeks in duration; however, longer-term data indicate that the initial symptomatic improvement from baseline with adjunctive brexpiprazole treatment is maintained in many patients.^47-49^ The studies did not include an adjunctive active comparator, which limits comparisons between agents, particularly given that most other atypical antipsychotics (with the exception of aripiprazole) have not been investigated in patients with minimal and partial response. Previous analyses have reported different responses to augmentation between males and females;^50^ there were not enough males to perform meaningful analyses by sex in the present sample. Finally, generalizability to a broader patient population is limited by patient selection criteria and restrictions. Future research should investigate whether patients with minimal response to antidepressant treatment obtain a larger benefit from initiating adjunctive brexpiprazole or switching to another antidepressant.

## CONCLUSION

Pooled clinical trial data showed greater improvements in depressive symptoms with adjunctive brexpiprazole versus adjunctive placebo in patients with minimal (>0% to <25%) response to antidepressant treatment and in patients with partial (≥25% to <50%) response to antidepressant treatment. Improvement was observed from the first week in patients with minimal response to antidepressant treatment. TEAEs in patients with minimal and partial response to antidepressant treatment aligned with those previously reported for the full sample. Practice patterns indicate that patients with minimal response to antidepressant treatment are often switched to another antidepressant. This analysis shows that clinicians could consider the alternative option of prescribing adjunctive brexpiprazole to patients with MDD and minimal response to their antidepressant treatment, as well as to patients who show a partial response to their antidepressant treatment.

## Supplementary Material

Supplement_3-Sep-25_pyaf074

## Data Availability

To submit inquiries related to Otsuka clinical research, or to request access to individual participant data (IPD) associated with any Otsuka clinical trial, please visit https://clinical-trials.otsuka.com/. For all approved IPD access requests, Otsuka will share anonymized IPD on a remotely accessible data sharing platform.

## References

[1] GBD 2021 Diseases and Injuries Collaborators . Global incidence, prevalence, years lived with disability (YLDs), disability-adjusted life-years (DALYs), and healthy life expectancy (HALE) for 371 diseases and injuries in 204 countries and territories and 811 subnational locations, 1990–2021: A systematic analysis for the Global Burden of Disease study 2021. *Lancet.* 2024;403:2133–2161. 38642570 10.1016/S0140-6736(24)00757-8 PMC11122111

[2] Rong J, Wang X, Cheng P, Li D, Zhao D. Global, regional and national burden of depressive disorders and attributable risk factors, from 1990 to 2021: Results from the 2021 Global Burden of Disease study. *Br J Psychiatry*. 2025;227:688-697. 10.1192/bjp.2024.266 39809717

[3] American Psychological Association . Clinical Practice Guideline for the Treatment of Depression across Three Age Cohorts. 2019. Accessed March 12, 2025. https://www.apa.org/depression-guideline.

[4] Lam RW, Kennedy SH, Adams C, et al. Canadian Network for Mood and Anxiety Treatments (CANMAT) 2023 update on clinical guidelines for management of major depressive disorder in adults. *Can J Psychiatr*. 2024;69:641–687. 10.1177/07067437241245384 PMC11351064 38711351

[5] National Institute for Health and Care Excellence. Depression in adults: Treatment and management. NICE guideline NG222 2022. Accessed December 2, 2024. https://www.nice.org.uk/guidance/ng222. 35977056

[6] Cipriani A, Furukawa TA, Salanti G, et al. Comparative efficacy and acceptability of 21 antidepressant drugs for the acute treatment of adults with major depressive disorder: A systematic review and network meta-analysis. *Lancet.* 2018;391:1357–1366. 10.1016/S0140-6736(17)32802-7 29477251 PMC5889788

[7] Papakostas GI, Fava M. Does the probability of receiving placebo influence clinical trial outcome? A meta-regression of double-blind, randomized clinical trials in MDD. *Eur Neuropsychopharmacol*. 2009;19:34–40. 10.1016/j.euroneuro.2008.08.009 18823760

[8] Mauskopf JA, Simon GE, Kalsekar A, et al. Nonresponse, partial response, and failure to achieve remission: Humanistic and cost burden in major depressive disorder. *Depress Anxiety*. 2009;26:83–97. 18833573 10.1002/da.20505

[9] Pastuszak M, Cubała WJ, Kwaśny A, Mechlińska A. The search for consistency in residual symptoms in major depressive disorder: A narrative review. *J Pers Med*. 2024;14:828. 10.3390/jpm14080828 39202019 PMC11355381

[10] Fava M, Davidson KG. Definition and epidemiology of treatment-resistant depression. *Psychiatr Clin North Am*. 1996;19:179–200. 10.1016/S0193-953X(05)70283-5 8827185

[11] Nierenberg AA, DeCecco LM. Definitions of antidepressant treatment response, remission, nonresponse, partial response, and other relevant outcomes: A focus on treatment-resistant depression. *J Clin Psychiatry.* 2001;62:5–9. 11480882

[12] Nelson JC, Thase ME, Bellocchio EE, et al. Efficacy of adjunctive aripiprazole in patients with major depressive disorder who showed minimal response to initial antidepressant therapy. *Int Clin Psychopharmacol*. 2012;27:125–133. 10.1097/YIC.0b013e3283502791 22466058

[13] Casey DE, Laubmeier KK, Eudicone JM, et al. Response and remission rates with adjunctive aripiprazole in patients with major depressive disorder who exhibit minimal or no improvement on antidepressant monotherapy. *Int J Clin Pract*. 2014;68:1301–1308. 10.1111/ijcp.12480 25196314

[14] Goldberg JF, Freeman MP, Balon R, et al. The American Society of Clinical Psychopharmacology survey of psychopharmacologists’ practice patterns for the treatment of mood disorders. *Depress Anxiety*. 2015;32:605–613. 10.1002/da.22378 26129956

[15] Han C, Wang SM, Kato M, et al. Second-generation antipsychotics in the treatment of major depressive disorder: Current evidence. *Expert Rev Neurother*. 2013;13:851–870. 23898855 10.1586/14737175.2013.811901

[16] Nuñez NA, Joseph B, Pahwa M, et al. Augmentation strategies for treatment resistant major depression: A systematic review and network meta-analysis. *J Affect Disord*. 2022;302:385–400. 10.1016/j.jad.2021.12.134 34986373 PMC9328668

[17] Kishimoto T, Hagi K, Kurokawa S, Kane JM, Correll CU. Efficacy and safety/tolerability of antipsychotics in the treatment of adult patients with major depressive disorder: A systematic review and meta-analysis. *Psychol Med*. 2023;53:4064–4082. 10.1017/S0033291722000745 35510505 PMC10317805

[18] Thase ME, Youakim JM, Skuban A, et al. Efficacy and safety of adjunctive brexpiprazole 2 mg in major depressive disorder: A phase 3, randomized, placebo-controlled study in patients with inadequate response to antidepressants. *J Clin Psychiatry*. 2015;76:1224–1231. 10.4088/JCP.14m09688 26301701

[19] Thase ME, Youakim JM, Skuban A, et al. Adjunctive brexpiprazole 1 and 3 mg for patients with major depressive disorder following inadequate response to antidepressants: A phase 3, randomized, double-blind study. *J Clin Psychiatry*. 2015;76:1232–1240. 10.4088/JCP.14m09689 26301771

[20] Hobart M, Skuban A, Zhang P, et al. A randomized, placebo-controlled study of the efficacy and safety of fixed-dose brexpiprazole 2 mg/d as adjunctive treatment of adults with major depressive disorder. *J Clin Psychiatry.* 2018;79:17m12058. 10.4088/JCP.17m12058 29873953

[21] Hobart M, Skuban A, Zhang P, et al. Efficacy and safety of flexibly dosed brexpiprazole for the adjunctive treatment of major depressive disorder: A randomized, active-referenced, placebo-controlled study. *Curr Med Res Opin*. 2018;34:633–642. 10.1080/03007995.2018.1430220 29343128

[22] Thase ME, Zhang P, Weiss C, Meehan SR, Hobart M. Efficacy and safety of brexpiprazole as adjunctive treatment in major depressive disorder: Overview of four short-term studies. *Expert Opin Pharmacother*. 2019;20:1907–1916. 10.1080/14656566.2019.1638913 31290344

[23] American Psychiatric Association . Diagnostic and Statistical Manual of Mental Disorders, Fourth Edition, Text Revision. Washington, DC: American Psychiatric Association, 2000.

[24] Chandler GM, Iosifescu DV, Pollack MH, Targum SD, Fava M. Validation of the Massachusetts General Hospital Antidepressant Treatment History Questionnaire (ATRQ). *CNS Neurosci Ther*. 2010;16:322–325. 10.1111/j.1755-5949.2009.00102.x 19769599 PMC6493891

[25] Hamilton M . A rating scale for depression. *J Neurol Neurosurg Psychiatry*. 1960;23:56–62. 10.1136/jnnp.23.1.56 14399272 PMC495331

[26] Guy W . ECDEU Assessment Manual for Psychopharmacology, Revised. Rockville, MD: National Institute of Mental Health, 1976.

[27] Montgomery SA, Åsberg M. A new depression scale designed to be sensitive to change. *Br J Psychiatry*. 1979;134:382–389. 444788 10.1192/bjp.134.4.382

[28] Otsuka Pharmaceutical Co. Ltd . Rexulti^®^ (brexpiprazole) tablets, for oral use. *Prescribing Information [United States]*. 2025. Accessed August 18, 2025. https://www.otsuka-us.com/media/static/Rexulti-PI.pdf

[29] Otsuka Pharmaceutical Co. Ltd . Rexulti^®^ brexpiprazole tablets. *Product Monograph [Canada]*. 2024. Accessed March 12, 2025. https://rexultimonograph.ca/

[30] Cohen J . A power primer. *Psychol Bull*. 1992;112:155–159. 10.1037/0033-2909.112.1.155 19565683

[31] Fava M, Rush AJ, Wisniewski SR, et al. A comparison of mirtazapine and nortriptyline following two consecutive failed medication treatments for depressed outpatients: A STAR^*^D report. *Am J Psychiatry*. 2006;163:1161–1172. 10.1176/ajp.2006.163.7.1161 16816220

[32] McGrath PJ, Stewart JW, Fava M, et al. Tranylcypromine versus venlafaxine plus mirtazapine following three failed antidepressant medication trials for depression: A STAR^*^D report. *Am J Psychiatry*. 2006;163:1531–1541. 10.1176/ajp.2006.163.9.1531 16946177

[33] Habert J, Katzman MA, Oluboka OJ, et al. Functional recovery in major depressive disorder: Focus on early optimized treatment. *Prim Care Companion CNS Disord*. 2016;18. 10.4088/PCC.15r01926 27835721

[34] Papakostas GI, Jackson WC, Rafeyan R, Trivedi MH. Inadequate response to antidepressant treatment in major depressive disorder. *J Clin Psychiatry.* 2020;81:OT19037COM5. 10.4088/JCP.OT19037COM5 32433833

[35] Fava M, Ménard F, Davidsen CK, Baker RA. Adjunctive brexpiprazole in patients with major depressive disorder and irritability: An exploratory study. *J Clin Psychiatry.* 2016;77:1695–1701. 10.4088/JCP.15m10470 27379823

[36] Davis LL, Ota A, Perry P, et al. Adjunctive brexpiprazole in patients with major depressive disorder and anxiety symptoms: An exploratory study. *Brain Behav*. 2016;6:e00520. 27781135 10.1002/brb3.520 PMC5064333

[37] McIntyre RS, Weiller E, Zhang P, Weiss C. Brexpiprazole as adjunctive treatment of major depressive disorder with anxious distress: Results from a *post-hoc* analysis of two randomised controlled trials. *J Affect Disord*. 2016;201:116–123. 10.1016/j.jad.2016.05.013 27208498

[38] Thase ME, Weiller E, Zhang P, Weiss C, McIntyre RS. Adjunctive brexpiprazole in patients with major depressive disorder and anxiety symptoms: *Post hoc* analyses of three placebo-controlled studies. *Neuropsychiatr Dis Treat*. 2019;15:37–45. 10.2147/NDT.S185815 30587996 PMC6305164

[39] McIntyre RS, Bubolic S, Zhang Z, et al. Effects of adjunctive brexpiprazole on individual depressive symptoms and functioning in patients with major depressive disorder and anxious distress: *Post hoc* analysis of three placebo-controlled studies. *J Clin Psychopharmacol*. 2024;44:133–140. 10.1097/JCP.0000000000001825 38421922

[40] Fava M, Weiller E, Zhang P, Weiss C. Efficacy of brexpiprazole as adjunctive treatment in major depressive disorder with irritability: *Post hoc* analysis of 2 pivotal clinical studies. *J Clin Psychopharmacol*. 2017;37:276–278. 10.1097/JCP.0000000000000678 28195853 PMC5325249

[41] Newcomer JW, Eriksson H, Zhang P, Meehan SR, Weiss C. Changes in metabolic parameters and body weight in patients with major depressive disorder treated with adjunctive brexpiprazole: Pooled analysis of phase 3 clinical studies. *J Clin Psychiatry.* 2019;80:18m12680. 10.4088/JCP.18m12680 31577867

[42] Newcomer JW, Meehan SR, Chen D, Brubaker M, Weiss C. Changes in metabolic parameters and body weight in patients with prediabetes treated with adjunctive brexpiprazole for major depressive disorder: Pooled analysis of short- and long-term clinical studies. *J Clin Psychiatry*. 2023;84:23m14786. 10.4088/JCP.23m14786 37656180

[43] Weiss C, Weiller E, Baker RA, et al. The effects of brexpiprazole and aripiprazole on body weight as monotherapy in patients with schizophrenia and as adjunctive treatment in patients with major depressive disorder: An analysis of short-term and long-term studies. *Int Clin Psychopharmacol*. 2018;33:255–260. 29878915 10.1097/YIC.0000000000000226 PMC6078484

[44] Clayton AH, Ivkovic J, Chen D, George V, Hobart M. Effect of brexpiprazole on prolactin and sexual functioning: An analysis of short- and long-term study data in major depressive disorder. *J Clin Psychopharmacol*. 2020;40:560–567. 10.1097/JCP.0000000000001297 33136923 PMC7643790

[45] Mohamed S, Johnson GR, Chen P, et al. Effect of antidepressant switching vs augmentation on remission among patients with major depressive disorder unresponsive to antidepressant treatment: The VAST-D randomized clinical trial. *JAMA.* 2017;318:132–145. 28697253 10.1001/jama.2017.8036 PMC5817471

[46] Oliva V, Vieta E. Predicting the past: The risks and rewards of post-hoc findings. *Eur Neuropsychopharmacol*. 2025;92:21–22. 10.1016/j.euroneuro.2024.12.005 39709733

[47] Bauer M, Hefting N, Lindsten A, Josiassen MK, Hobart M. A randomised, placebo-controlled 24-week study evaluating adjunctive brexpiprazole in patients with major depressive disorder. *Acta Neuropsychiatr*. 2019;31:27–35. 10.1017/neu.2018.23 30223911

[48] Hobart M, Zhang P, Skuban A, et al. A long-term, open-label study to evaluate the safety and tolerability of brexpiprazole as adjunctive therapy in adults with major depressive disorder. *J Clin Psychopharmacol*. 2019;39:203–209. 10.1097/JCP.0000000000001034 30946704 PMC6494030

[49] McIntyre RS, Sundararajan K, Behl S, et al. A double-blind, placebo-controlled, randomised withdrawal study of adjunctive brexpiprazole maintenance treatment for major depressive disorder. *Acta Neuropsychiatr*. 2024;37:e33. 10.1017/neu.2024.32 PMC13130322 39415650

[50] Moderie C, Nuñez N, Fielding A, Comai S, Gobbi G. Sex differences in responses to antidepressant augmentations in treatment-resistant depression. *Int J Neuropsychopharmacol*. 2022;25:479–488. 10.1093/ijnp/pyac017 35167671 PMC9211005

