# Supplementary material for: Adjunctive brexpiprazole in patients with major depressive disorder who show minimal or partial response to antidepressant treatment: post hoc analysis of randomized controlled trials
Source: Int J Neuropsychopharmacol. 2025 Oct 7;28(10):pyaf074. doi: 10.1093/ijnp/pyaf074 (PMC12554155; doi:10.1093/ijnp/pyaf074)
Supplement: Supplement_3-Sep-25_pyaf074 [file supplement_3-sep-25_pyaf074.pdf]

Supplementary Material

Adjunctive Brexpiprazole in Patients With Major Depressive Disorder Who Show Minimal or Partial Response to Antidepressant Treatment: *Post Hoc* Analysis of Randomized Controlled Trials

Shivani Kapadia, PharmD,[a] Zhen Zhang, PhD,[b] Ferhat Ardic, MD,[c] Mehul Patel, PharmD,[a] Michael E. Thase, MD,[d] George I. Papakostas, MD[e]

[a]Medical Affairs, Otsuka Pharmaceutical Development & Commercialization Inc., Princeton, NJ, USA
[b]Medical and Real-World Data Analytics, Otsuka Pharmaceutical Development & Commercialization Inc., Princeton, NJ, USA
[c]Medical Affairs, H. Lundbeck A/S, Valby, Denmark
[d]Perelman School of Medicine, University of Pennsylvania and the Philadelphia Veterans Affairs Medical Center, Philadelphia, PA, USA
[e]Department of Psychiatry, Clinical Trials Network and Institute, Massachusetts General Hospital, Harvard Medical School, Boston, MA, USA

Contents – Results for 2 mg analyses

Patients **2**
Minimal (0–25%) Response to ADT Subgroup 2
Partial (25–50%) Response to ADT Subgroup 2
Efficacy **2**
Minimal (0–25%) Response to ADT Subgroup 2
Partial (25–50%) Response to ADT Subgroup 3
Safety **3**
Minimal (0–25%) Response to ADT Subgroup 3
Partial (25–50%) Response to ADT Subgroup 3
Supplementary Table S1. Baseline characteristics and assigned ADTs for ADT + brexpiprazole 2 mg and ADT + placebo, stratified by response to ADT 4
Supplementary Table S2. Summary of TEAEs for ADT + brexpiprazole 2 mg and ADT + placebo, stratified by response to ADT 6
Supplementary Figure S1. Mean MADRS total score change for ADT + brexpiprazole 2 mg versus ADT + placebo, stratified by response to ADT 7

Patients

Across the two trials, the efficacy sample comprised 746 patients. The ADTs assigned to subgroups of these patients are listed in Supplementary Table S1. Over 8 weeks in the prospective ADT period, 177 (23.7%) patients did not improve (≤0%) on ADT, 415 (55.6%) patients had minimal (0–25%) response to ADT, and 154 (20.6%) patients had partial (25–50%) response to ADT.

Minimal (0–25%) Response to ADT Subgroup

Following randomization, the minimal response to ADT subgroup comprised 201 patients on ADT + brexpiprazole 2 mg/day and 214 patients on ADT + placebo. The 6-week randomized treatment period was completed by 190 (94.5%) patients on ADT + brexpiprazole 2 mg/day and 206 (96.3%) patients on ADT + placebo.

At baseline (ie, the start of the randomized treatment period), mean (SD) MADRS total score was 26.5 (4.4) for ADT + brexpiprazole 2 mg/day and 26.8 (4.6) for ADT + placebo. These scores had improved from 30.5 (4.5) and 30.7 (5.1), respectively, at the start of the 8-week prospective ADT period.

The minimal response to ADT subgroup was 70.4% (292/415) female, 29.6% (123/415) male, 88.2% (366/415) White, and 11.8% (49/415) other races (including American Indian or Alaska Native, Asian, Black or African American, Native Hawaiian or Other Pacific Islander, and other non-specified). Further demographic and clinical characteristics at baseline of the randomized treatment period are presented in Supplementary Table S1, and were similar between treatments.

Partial (25–50%) Response to ADT Subgroup

Following randomization, the partial response to ADT subgroup comprised 70 patients on ADT + brexpiprazole 2 mg/day and 84 patients on ADT + placebo. The 6-week randomized treatment period was completed by 64 (91.4%) patients on ADT + brexpiprazole 2 mg/day and 81 (96.4%) patients on ADT + placebo.

At baseline (ie, the start of the randomized treatment period), mean (SD) MADRS total score was 21.6 (3.9) for ADT + brexpiprazole 2 mg/day and 20.9 (3.5) for ADT + placebo. These scores had improved from 31.3 (5.0) and 30.9 (4.0), respectively, at the start of the 8-week prospective ADT period.

The partial response to ADT subgroup was 70.1% (108/154) female, 29.9% (46/154) male, 84.4% (130/154) White, and 15.6% (24/154) other races. Further demographic and clinical characteristics at baseline of the randomized treatment period are presented in Supplementary Table S1, and were similar between treatments.

Efficacy

Minimal (0–25%) Response to ADT Subgroup

In patients with minimal response to ADT, LS mean (SE) MADRS total score change from baseline to Week 6 was -9.0 (0.6) points for ADT + brexpiprazole 2 mg/day and -7.0 (0.6) points for ADT + placebo. The LS mean (95% CI) treatment difference at Week 6 was -1.98 (-3.56 to -0.40), with $p=0.014$ and effect size 0.24 in favor of ADT + brexpiprazole 2 mg/day. Considering other timepoints, ADT + brexpiprazole 2 mg/day was associated with greater improvement than ADT + placebo ($p<0.05$) at Week 1 and from Week 3, onwards (Supplementary Figure S1A).

CGI-S results were supportive, with LS mean (SE) changes from baseline to Week 6 of -1.1 (0.1) for ADT + brexpiprazole 2 mg/day and -0.9 (0.1) for ADT + placebo, with an

LS mean (95% CI) treatment difference of -0.23 (-0.38 to -0.09); p=0.002 and effect size 0.30.

Partial (25–50%) Response to ADT Subgroup

In patients with partial response to ADT, LS mean (SE) MADRS total score change from baseline to Week 6 was -6.8 (0.7) points for ADT + brexpiprazole 2 mg/day and -4.7 (0.6) points for ADT + placebo. The LS mean (95% CI) treatment difference at Week 6 was -2.08 (-3.90 to -0.25), with p=0.026 and effect size 0.36 in favor of ADT + brexpiprazole 2 mg/day. Considering other timepoints, ADT + brexpiprazole 2 mg/day was associated with greater improvement than ADT + placebo (p<0.05) from Week 4, onwards (Supplementary Figure S1B).

CGI-S results were supportive, with LS mean (SE) changes from baseline to Week 6 of -1.1 (0.1) for ADT + brexpiprazole 2 mg/day and -0.8 (0.1) for ADT + placebo, with an LS mean (95% CI) treatment difference of -0.28 (-0.53 to -0.03); p=0.030 and effect size 0.35.

Safety

Minimal (0–25%) Response to ADT Subgroup

In patients with minimal response to ADT, the incidence of TEAEs was 118/201 (58.7%) for ADT + brexpiprazole 2 mg/day and 100/214 (46.7%) for ADT + placebo. TEAEs with incidence >5% for ADT + brexpiprazole 2 mg/day and greater than ADT + placebo were akathisia, increased weight, and restlessness; details are presented in Supplementary Table S2.

Partial (25–50%) Response to ADT Subgroup

In patients with partial response to ADT, the incidence of TEAEs was 38/70 (54.3%) for ADT + brexpiprazole 2 mg/day and 37/84 (44.0%) for ADT + placebo. TEAEs with incidence >5% for ADT + brexpiprazole 2 mg/day and greater than ADT + placebo were akathisia, increased weight, and nasopharyngitis; details are presented in Supplementary Table S2.

Supplementary Table S1. Baseline characteristics and assigned ADTs for ADT + brexpiprazole 2 mg and ADT + placebo, stratified by response to ADT

Characteristic, n (%) unless otherwise stated	Minimal (0–25%) response to ADT subgroup		Partial (25–50%) response to ADT subgroup	
	ADT + placebo (n=214)	ADT + brexpiprazole 2 mg (n=201)	ADT + placebo (n=84)	ADT + brexpiprazole 2 mg (n=70)
Age (years), mean (SD)	43.6 (12.5)	43.9 (11.7)	43.7 (11.8)	44.0 (12.0)
Sex				
Female	152 (71.0)	140 (69.7)	55 (65.5)	53 (75.7)
Male	62 (29.0)	61 (30.3)	29 (34.5)	17 (24.3)
BMI (kg/m^2), mean (SD)	28.8 (6.8)	29.4 (6.6)	30.3 (7.5)	30.0 (7.4)
Race				
White	187 (87.4)	179 (89.1)	71 (84.5)	59 (84.3)
Other[a]	27 (12.6)	22 (10.9)	13 (15.5)	11 (15.7)
Duration of current episode (months), mean (SD)	14.7 (27.8)	13.5 (14.4)	14.5 (19.3)	12.5 (14.7)
Number of lifetime episodes, mean (SD)	3.4 (2.8)	3.5 (2.8)	3.7 (2.3)	3.1 (2.1)
Number of prior ADTs at screening[b]				
1	184 (86.0)	165 (83.3)	65 (77.4)	53 (77.9)
2	29 (13.6)	30 (15.2)	14 (16.7)	13 (19.1)
3	1 (0.5)	3 (1.5)	5 (6.0)	2 (2.9)
MADRS total score, mean (SD)	26.8 (4.6)	26.5 (4.4)	20.9 (3.5)	21.6 (3.9)
CGI-S score, mean (SD)	4.3 (0.6)	4.3 (0.6)	3.9 (0.6)	3.8 (0.5)
Assigned ADT				
Escitalopram	43 (20.1)	37 (18.4)	14 (16.7)	19 (27.1)
Fluoxetine	32 (15.0)	36 (17.9)	10 (11.9)	4 (5.7)

Characteristic, n (%) unless otherwise stated	Minimal (0–25%) response to ADT subgroup		Partial (25–50%) response to ADT subgroup	
	ADT + placebo (n=214)	ADT + brexpiprazole 2 mg (n=201)	ADT + placebo (n=84)	ADT + brexpiprazole 2 mg (n=70)
Paroxetine CR	25 (11.7)	21 (10.4)	13 (15.5)	10 (14.3)
Sertraline	31 (14.5)	34 (16.9)	18 (21.4)	11 (15.7)
Duloxetine	49 (22.9)	38 (18.9)	17 (20.2)	11 (15.7)
Venlafaxine XR	34 (15.9)	35 (17.4)	12 (14.3)	15 (21.4)

[a]Including American Indian or Alaska Native, Asian, Black or African American, Native Hawaiian or Other Pacific Islander, and other non-specified.

[b]Number of prior ADTs at screening was missing for 5 patients.

Abbreviations: ADT, antidepressant treatment; BMI, body mass index; CGI-S, Clinical Global Impressions – Severity of illness; CR, controlled release; MADRS, Montgomery–Åsberg Depression Rating Scale; SD, standard deviation; XR, extended release.

Supplementary Table S2. Summary of TEAEs for ADT + brexpiprazole 2 mg and ADT + placebo, stratified by response to ADT

Event, n (%)	Minimal (0–25%) response to ADT subgroup		Partial (25–50%) response to ADT subgroup	
	ADT + placebo (n=214)	ADT + brexpiprazole 2 mg (n=201)	ADT + placebo (n=84)	ADT + brexpiprazole 2 mg (n=70)
At least 1 TEAE	100 (46.7)	118 (58.7)	37 (44.0)	38 (54.3)
Discontinued due to adverse event	1 (0.5)	6 (3.0)	0	0
At least 1 EPS-related TEAE	13 (6.1)	25 (12.4)	2 (2.4)	10 (14.3)
TEAEs with incidence >5% for ADT + brexpiprazole and greater than ADT + placebo				
Akathisia	9 (4.2)	16 (8.0)	0	6 (8.6)
Increased weight	6 (2.8)	12 (6.0)	0	4 (5.7)
Nasopharyngitis	7 (3.3)	5 (2.5)	3 (3.6)	4 (5.7)
Restlessness	2 (0.9)	12 (6.0)	0	3 (4.3)

Abbreviations: ADT, antidepressant treatment; EPS, extrapyramidal symptom; TEAE, treatment-emergent adverse event.

Supplementary Figure S1. Mean MADRS total score change for ADT + brexpiprazole 2 mg versus ADT + placebo, stratified by response to ADT

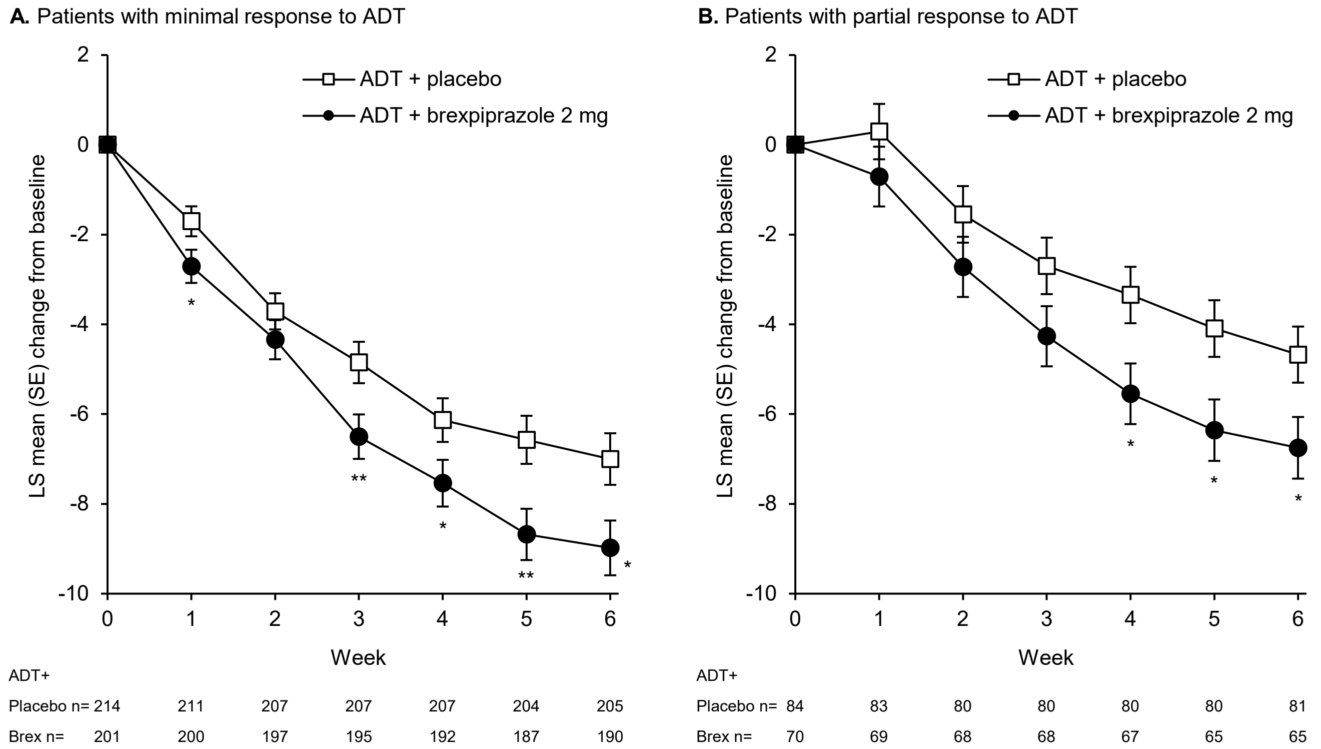

*p<0.05, **p<0.01 versus ADT + placebo; MMRM, observed cases.

Mean baseline scores (ADT + placebo, ADT + brexpiprazole 2 mg): A) minimal response: 26.8, 26.5; B) partial response: 20.9, 21.6.

Abbreviations: ADT, antidepressant treatment; Brex, brexpiprazole; LS, least squares; MADRS, Montgomery–Åsberg Depression Rating Scale; MMRM, mixed model for repeated measures; SE, standard error.
